# Patients with type 1 and type 2 diabetes hospitalized with COVID-19 in comparison with influenza: mortality and cardiorenal complications assessed by nationwide Swedish registry data

**DOI:** 10.1186/s12933-022-01719-x

**Published:** 2022-12-15

**Authors:** Robin Kristófi, Johan Bodegard, Viveca Ritsinger, Marcus Thuresson, David Nathanson, Thomas Nyström, Anna Norhammar, Jan W. Eriksson

**Affiliations:** 1 Department of Medical Sciences, Clinical Diabetes and Metabolism, Uppsala University, 75185 Uppsala, Sweden; 2 Cardiovascular, Renal and Metabolism, Medical Department, BioPharmaceuticals, AstraZeneca, Oslo, Norway; 3 Cardiology Unit, Department of Medicine, Karolinska Institute, Solna, Stockholm, Sweden; 4 Department of Research and Development, Region Kronoberg, Växjö, Sweden; 5 Statisticon AB, Uppsala, Sweden; 6 Department of Medicine, Karolinska Institutet, Huddinge, Stockholm, Sweden; 7 Department of Clinical Science and Education, Karolinska Institutet, Södersjukhuset, Stockholm, Sweden; 8 Capio St. Göran Hospital, Stockholm, Sweden

**Keywords:** Coronavirus 2019 (COVID-19), Type 1 diabetes, Type 2 diabetes, Mortality, Cardiovascular complications

## Abstract

**Background:**

The risk of severe coronavirus disease 2019 (COVID-19) is increased in people with diabetes, but effects of diabetes type and other risk factors remain incompletely characterized. We studied this in a Swedish cohort of hospitalized patients with type 1 and type 2 diabetes (T1D and T2D), also including comparisons with influenza epidemics of recent years.

**Methods:**

Nationwide healthcare registries were used to identify patients. A total of 11,005 adult patients with diabetes (T1D, n = 373; T2D, n = 10,632) were hospitalized due to COVID-19 from January 1, 2020 to September 1, 2021. Moreover, 5111 patients with diabetes (304 T1D, 4807 T2D) were hospitalized due to influenza from January 1, 2015 to December 31, 2019. Main outcomes were death within 28 days after admission and new hospitalizations for heart failure (HF), chronic kidney disease (CKD), cardiorenal disease (CRD; composite of HF and CKD), myocardial infarction (MI) and stroke during 1 year of follow-up.

**Results:**

Number of deaths and CRD events were 2025 and 442 with COVID-19 and 259 and 525 with influenza, respectively. Age- and sex-adjusted Cox regression models in COVID-19 showed higher risk of death and HF in T1D vs. T2D, hazard ratio (HR) 1.77 (95% confidence interval 1.41–2.22) and 2.57 (1.31–5.05). With influenza, T1D was associated with higher risk of death compared with T2D, HR 1.80 (1.26–2.57). Older age and previous CRD were associated with higher risks of death and hospitalization for CRD. After adjustment for prior comorbidities, mortality differences were still significant, but there were no significant differences in cardiovascular and renal outcomes. COVID-19 relative to influenza was associated with higher risk of death in both T1D and T2D, HR 2.44 (1.60–3.72) and 2.81 (2.59–3.06), respectively.

**Conclusions:**

In Sweden, patients with T1D as compared to T2D had a higher age- and sex-adjusted risk of death within 28 days and HF within one year after COVID-19 hospitalization, whereas the risks of other non-fatal cardiovascular and renal disease events were similar. Patients with T1D as well as T2D have a greater mortality rate when hospitalized due to COVID-19 compared to influenza, underscoring the importance of vaccination and other preventive measures against COVID-19 for diabetes patients.

**Supplementary Information:**

The online version contains supplementary material available at 10.1186/s12933-022-01719-x.

## Introduction

After the appearance of the severe acute respiratory syndrome coronavirus 2 (SARS-CoV-2) in December 2019 in Wuhan, China, the virus rapidly spread across the world, prompting the World Health Organization (WHO) to declare a pandemic in March 2020 [1]. At the time of writing, the WHO had registered around 298 million cases and 5.4 million deaths worldwide since the start of the pandemic [2]. The clinical manifestations of the disease caused by SARS-CoV-2 (COVID-19) vary widely, from asymptomatic infection to severe respiratory failure with multi-organ dysfunction and death [3]. The risk of severe disease is greatly influenced by the presence of comorbidities, and diabetes has been shown to be one of the most important conditions contributing to excess risk of severe disease [4–7]. It is important to better understand the risk factors for severe disease, in particular in vulnerable patient groups such as people with diabetes. Furthermore, few studies have directly compared the impact of COVID-19 on patients with type 1 (T1D) and type 2 diabetes (T2D).

We aimed to describe the characteristics of patients with diabetes in Sweden who were hospitalized with COVID-19 and to compare the two major types of diabetes with respect to risk for complications, in particular mortality and cardiovascular and renal disease events. The underlying risk factors for severe disease were also addressed. We also aimed to compare the complications of COVID-19 with those of the influenza epidemics of previous years as both are respiratory viruses capable of causing severe disease.

## Methods

This work is part of the CARE-19 study programme (Characteristics, Risks and Complications of Hospitalized COVID-19 Patients: Short- and Long-Term Observational Study) and based on the DAISY project to investigate the complications and treatment of diabetes. The overall aim of CARE-19 is to investigate and describe the direct and indirect effects of the COVID-19 pandemic on patients with cardiorenal diseases and diabetes. The study was approved by the Stockholm Regional Ethics Committee (reference numbers 2020-02917 and 2013/2206-31).

### Data sources

Data were retrieved from Swedish national registries. Sweden has a comprehensive, nationwide public health system and all citizens have a unique personal identification number (PIN) that is used for administrative purposes, including all contact with the healthcare system. Individual patient-level data from the Prescribed Drug Register (PDR), Cause of Death Register (CoDR) and National Patient Register (NPR) were linked with the PIN in a pseudonymized manner. The PDR contains information on all expedited prescriptions by Swedish pharmacies, as they are bound by law to report these to the Swedish eHealth Agency which manages this registry. The NPR contains data on all in- and outpatient secondary (i.e. hospital) care involving a physician in Sweden, with coverage being nearly universal. The CoDR records the cause of death of deceased persons in Sweden, as reported by the physician who confirmed the death. Information regarding cause of death is based on the clinical details surrounding the death, prior medical history and autopsy results, if applicable. The latter two registries are managed by the Swedish National Board of Health and Welfare.

### Study population

All adult patients > 18 years of age with T1D or T2D in Sweden who had been hospitalized due to COVID-19 between January 1, 2020 and September 9, 2021 or due to influenza between January 1, 2015 and December 31, 2019 were included in the study population. The time span for influenza was chosen because cases dropped sharply with the advent of the COVID-19 pandemic at the beginning of 2020, and the preceding years represented average influenza seasons. Patients were identified using the PDR and NPR. COVID-19 and influenza hospitalization were defined according to a main ICD-10 diagnosis code of COVID-19 or influenza, respectively, at discharge. The index date was chosen to be the date of admission. T1D was defined as having received at least one prescription of a short-acting insulin from the start of treatment or within 6 months of starting basal insulin treatment and having a diagnosis of T1D in secondary care. We have previously shown this definition to be a valid and accurate way to identify T1D patients in Sweden [8]. T2D was defined as having had any glucose-lowering drug dispensed by a pharmacy, with the exception of those defined as T1D and those with a diagnosis of gestational diabetes or polycystic ovary syndrome. Baseline comorbidities were retrieved from the NPR prior to the index date stretching back to 1998. ICD-10 and surgical codes of baseline comorbidities are presented in Additional file 1: Table S1.

### Outcomes

Outcomes were retrieved from the NPR. Diagnosis codes from ICD-10 were used to define outcomes, and only the first diagnosis position as main diagnosis was considered (for ICD-10 codes, see Additional file 1: Table S2). Subjects were followed until death or up to 1 year after admission. The following outcomes were assessed: all-cause death and hospitalization due to non-fatal heart failure (HF), chronic kidney disease (CKD), cardiorenal disease (CRD; defined as a composite of HF or CKD), myocardial infarction (MI), and stroke. Death rate was assessed during the first 28 days from admission. Other outcomes were defined as having occurred within up to 1 year of follow-up after admission. Time to first event was used in the regression models and not repeated events of the same kind. Mean follow-up ranged from 6.9 to 8.1 in COVID-19 and 9.7 to 10.5 months in influenza, according to outcome and type of diabetes (Additional file 1: Table S3).

### Statistical analyses

Cox proportional hazards regression models were used to determine the hazard ratio (HR) and 95% confidence interval of various risk factors on outcomes. Analyses of outcomes were performed with 1 year follow-up after the index date, except for death (first 28 days). Risk factors included type of diabetes, age, female sex, HF, CKD, ischemic heart disease, stroke, peripheral artery disease, previous pneumonia, chronic obstructive pulmonary disease (COPD), obesity and rheumatologic disease defined according to ICD-10 codes. Ischemic heart disease was defined as any of the following: myocardial infarction, unstable angina, angina pectoris and PCI and CABG procedures. In addition to fully adjusted models, we also did regression models only adjusting for age and sex, as well as a subgroup analysis in subjects without previous cardiovascular or renal disease at baseline.

## Results

### Baseline data

A total of 11,005 patients with diabetes hospitalized with COVID-19 were included (373 patients with T1D and 10,632 patients with T2D), Table 1. The corresponding figure for influenza was 5111 patients with diabetes (304 patients with T1D and 4807 patients with T2D). Patients with T1D were on average younger than T2D patients and age distribution was similar in both COVID-19 and influenza (mean ages 58 and 57 years for T1D; 70 and 76 years for T2D). Median duration of in-hospital stay was slightly shorter in the case of influenza than in COVID-19 (5 vs. 8 days). In general, hospitalized T1D and T2D patients with both COVID-19 and influenza had a high comorbidity burden. For instance, in COVID-19, around 20% of patients had heart failure and in influenza, around 30% of T1D and 40% of T2D patients had ischemic heart disease.

**Table 1:** Baseline characteristics including previous medical history of patients with diabetes hospitalized due to COVID-19 or influenza

	COVID-19 hospitalizations years 2020–2021			Influenza hospitalizations years 2015–2019		
	Diabetes	T1D	T2D	Diabetes	T1D	T2D
Number of patients, n (%)	11,005	373 (3)	10,632 (97)	5111	304 (6)	4807 (94)
Age (years), mean (SD)	69 (13)	58 (17)	70 (13)	75 (12)	57 (16)	76 (11)
Females, n (%)	4136 (38)	134 (36)	4002 (38)	2220 (43)	137 (45)	2083 (43)
Hospitalization duration, median days (IQR; range)	8 (4–13; 1–425)	6 (3–11; 1–102)	8 (4–13; 1–425)	5 (3–9; 1–654)	4 (3–7; 1–64)	5 (3–9; 1–654)
Heart failure, n (%)	2075 (19)	79 (21)	1996 (19)	1526 (30)	63 (21)	1463 (30)
CKD, n (%)	1389 (13)	84 (23)	1305 (12)	744 (15)	80 (26)	664 (14)
Dialysis, n (%)	311 (3)	46 (12)	265 (2)	187 (4)	45 (15)	142 (3)
Ischemic heart disease, n (%)	3242 (29)	99 (27)	3143 (30)	1970 (39)	95 (31)	1875 (39)
Stroke, n (%)	1953 (18)	58 (16)	1895 (18)	1256 (25)	48 (16)	1208 (25)
Atrial fibrillation, n (%)	2115 (19)	49 (13)	2066 (19)	1506 (29)	33 (11)	1473 (31)
Peripheral artery disease, n (%)	740 (7)	57 (15)	683 (6)	516 (10)	60 (20)	456 (9)
COPD, n (%)	1069 (10)	34 (9)	1035 (10)	774 (15)	20 (7)	754 (16)
Obesity, n (%)	1911 (17)	65 (17)	1846 (17)	729 (14)	22 (7)	707 (15)
Rheumatologic disease, n (%)	1064 (10)	40 (11)	1024 (10)	617 (12)	33 (11)	584 (12)
Pneumonia, n (%)	2418 (22)	107 (29)	2311 (22)	1793 (35)	123 (40)	1,670 (35)
Thromboembolism (DVT or PE), n (%)	849 (8)	32 (9)	817 (8)	497 (10)	23 (8)	474 (10)
Cancer, n (%)	2505 (23)	55 (15)	2450 (23)	1511 (30)	53 (17)	1458 (30)

Data are n (%), mean (*SD* standard deviation) or median (*IQR* interquartile range; range) as indicated. *CKD* chronic kidney disease. *COPD* chronic obstructive pulmonary disease. *DVT* deep vein thrombosis. *PE* pulmonary embolism

### Outcomes

#### Number of events in T1D and T2D in COVID-19 and influenza

In the first 28 days after admission, there were 2025 deaths (mortality 18.4%) in the COVID-19 group, of which 1962 were T2D and 63 T1D patients (mortality 18.5% and 16.9%, respectively). In the influenza group, 247 deaths occurred (mortality 4.8%), of which 237 were among T2D and 10 among T1D patients (mortality 4.9% and 3.3%, respectively). 60-day mortality was 20.9% for T2D and 19.0% for T1D. This was higher in 2020 (23.6% T2D, 21.8% T1D) than in 2021 (16.2% T2D, 12.6% T1D; see Additional file 1: Table S4). Over the follow-up period of up to 1 year after admission, there were 229 cases of incident HF in COVID-19 (9 T1D, 220 T2D; 2.4 vs. 2.1%) and 353 cases in influenza (13 T1D, 340 T2D; 4.3 vs. 7.1%). For CKD, the corresponding figures were 234 in COVID-19 (8 T1D, 226 T2D; 2.1 vs. 2.1%) and 201 in influenza (20 T1D, 181 T2D; 6.6 vs. 3.8%). There were 63 cases of MI in the COVID-19 group (3 T1D, 60 T2D), and 85 cases in the influenza group (5 T1D, 80 T2D). For stroke, there were 97 cases in COVID-19 (4 T1D, 93 T2D) and 98 cases in influenza (5 T1D, 93 T2D). The number of events and event rates for each outcome are presented in Additional file 1: Table S5.

#### Incidence of events during one-year follow-up

**Fig. 1:**
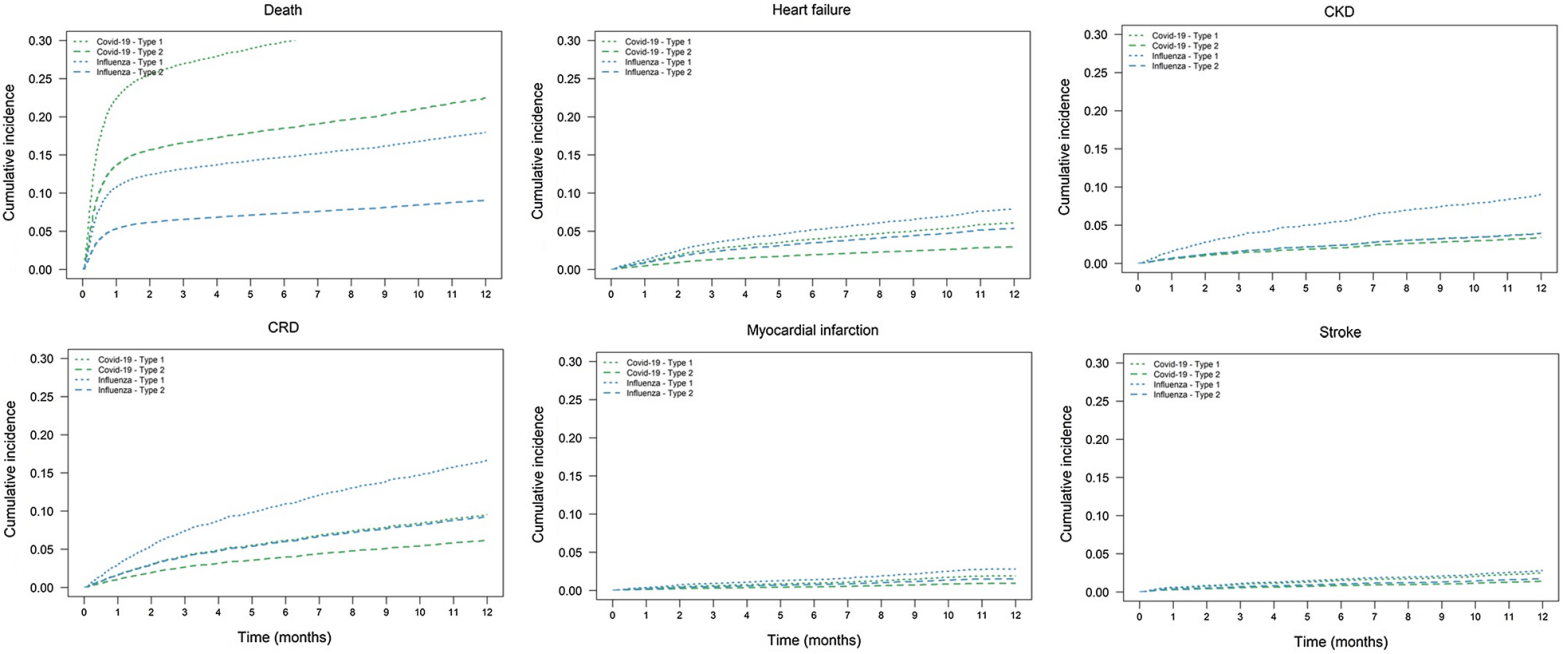
Age- and sex-adjusted cumulative incidence of events following hospitalization due to COVID-19 or influenza

The age- and sex-adjusted cumulative incidence of outcomes up to 1 year after admission is presented in Fig. 1. In T2D, the incidence of HF was higher after influenza vs COVID-19 hospitalization, and it was higher in T1D compared to T2D following COVID-19 (Table 2). Mortality was clearly  higher in COVID-19 relative to influenza, and in T1D relative to T2D. Deaths occurred at a greater rate in the first 1–2 months after admission than during the rest of the follow-up period.

#### Risk of mortality and morbidity in T1D vs. T2D

Cox regression models adjusted for age and sex in COVID-19 showed higher risks of mortality and HF in T1D vs. T2D, with HR values 1.77 (95% CI 1.41–2.22) and 2.57 (95% CI 1.31–5.05), respectively, Table 2. However, there were no significant differences in any of the other outcomes. In the equivalent models for influenza, T1D was associated with higher risk of death with HR 1.80 (95% CI 1.26–2.57). There were no significant differences among the cardiorenal outcomes, although there was a near-significant tendency to higher risk of CKD in T1D, HR 1.43 (0.97–2.11).

**Table 2:** Age- and sex-adjusted risks (hazard ratios) of death within 28 days and cardiorenal complications within 1 year from admission, in relation to baseline conditions

	COVID-19 (11,005 hospitalized patients during 2020–2021)					
	Death	HF	CKD	CRD	MI	Stroke
Number of events	2025	229	234	442	63	97
T1D vs. T2D	1.77 (1.41–2.22)	2.57 (1.31–5.05)	1.40 (0.68–2.85)	1.83 (1.10–3.03)	2.30 (0.71–7.46)	1.86 (0.67–5.12)
Age (per 10-year increase)	2.17 (2.08–2.25)	2.32 (2.03–2.64)	1.40 (1.26–1.56)	1.73 (1.59–1.89)	1.64 (1.32–2.05)	1.53 (1.29–1.83)
Female sex	0.76 (0.70–0.83)	0.73 (0.56–0.96)	0.84 (0.64–1.10)	0.78 (0.64–0.95)	1.00 (0.60–1.67)	0.83 (0.55–1.26)

	Influenza (5111 hospitalized patients during 2015–2019)					
	Death	HF	CKD	CRD	MI	Stroke
Number of events	247	353	201	525	85	98
T1D vs. T2D	1.80 (1.26–2.57)	1.15 (0.65–2.05)	1.60 (0.95–2.68)	1.43 (0.97–2.11)	1.59 (0.61–4.16)	1.50 (0.58–3.88)
Age (per 10-year increase)	1.86 (1.72–2.01)	1.49 (1.34–1.66)	0.96 (0.86–1.08)	1.24 (1.14–1.35)	1.34 (1.08–1.65)	1.42 (1.16–1.74)
Female sex	0.92 (0.80–1.06)	1.00 (0.81–1.24)	0.99 (0.75–1.31)	0.98 (0.82–1.16)	0.72 (0.47–1.13)	0.86 (0.58–1.29)

Diabetes patients with type 1 (T1D) or type 2 diabetes (T2D) hospitalized for Covid-19 or influenza. Risks were estimated with age- and sex-adjusted hazard ratio values including 95% confidence interval

#### Variables associated with increased mortality risk

In fully adjusted Cox regression models including comorbidities, risk of death following COVID-19 hospitalization was greater in T1D than in T2D (HR 1.44, 95% CI 1.15–1.81), Table 3. Mortality from influenza was also increased in T1D vs. T2D (HR 1.62, 95% CI 1.12–2.33). In the COVID-19 cohort, age emerged as the strongest risk factor for death, with prior diagnosis of HF, CKD, stroke, peripheral artery disease and pneumonia also portending a poorer prognosis. Female sex was associated with a lower mortality risk, whereas ischemic heart disease, obesity and rheumatologic disease were neutral in this regard. In the influenza cohort, female sex was not, whereas rheumatologic disease was associated with higher risk, but otherwise findings were similar to the COVID-19 cohort.

**Table 3:** Fully adjusted risks (hazard ratios) of death within 28 days and cardiorenal complications within 1 year after admission, in relation to baseline conditions

	COVID-19 (11,005 hospitalized patients during 2020–2021)					
	Death	HF	CKD	CRD	MI	Stroke
Number of events	2025	229	234	442	63	97
T1D vs. T2D	1.44 (1.15–1.81)	1.56 (0.78–3.10)	0.79 (0.38–1.63)	1.11 (0.66–1.85)	1.90 (0.58–6.28)	1.65 (0.59–4.60)
Age (per 10-year increase)	1.97 (1.89–2.06)	1.70 (1.47–1.97)	1.21 (1.07–1.36)	1.38 (1.26–1.52)	1.39 (1.09–1.78)	1.33 (1.10–1.61)
Female sex	0.80 (0.74–0.87)	0.76 (0.57–1.00)	0.91 (0.69–1.20)	0.81 (0.66–0.99)	1.12 (0.67–1.88)	0.92 (0.60–1.41)
HF	1.27 (1.15–1.40)	8.73 (5.52–7.15)	1.35 (0.97–1.88)	3.49 (2.76–4.41)	1.81 (0.98–3.37)	1.06 (0.61–1.85)
CKD	1.56 (1.42–1.72)	1.58 (1.18–2.12)	6.03 (4.50–8.06)	3.05 (2.47–3.77)	1.18 (0.59–2.38)	1.41 (0.79–2.52)
Ischemic heart disease	1.04 (0.96–1.13)	1.13 (0.85–1.50)	0.84 (0.63–1.14)	0.95 (0.77–1.17)	1.92 (1.11–3.33)	1.16 (0.74–1.83)
Stroke	1.16 (1.07–1.27)	0.85 (0.63–1.15)	1.02 (0.74–1.41)	0.93 (0.74–1.17)	1.43 (0.81–2.53)	2.62 (1.70–4.05)
Peripheral artery disease	1.38 (1.22–1.55)	0.98 (0.67–1.45)	1.13 (0.73–1.74)	0.95 (0.70–1.29)	0.83 (0.32–2.14)	0.93 (0.42–2.05)
Pneumonia	1.36 (1.25–1.48)	1.10 (0.83–1.46)	1.46 (1.10–1.95)	1.29 (1.05–1.59)	0.84 (0.45–1.57)	0.99 (0.60–1.63)
COPD	1.08 (0.97–1.21)	1.35 (0.99–1.84)	0.87 (0.58–1.31)	1.15 (0.90–1.48)	0.66 (0.28–1.59)	0.68 (0.32–1.43)
Obesity	1.06 (0.95–1.18)	1.44 (1.06–1.96)	1.13 (0.82–1.56)	1.27 (1.02–1.60)	0.78 (0.37–1.62)	0.48 (0.23–1.00)
Rheumatologic disease	0.97 (0.86–1.09)	1.17 (0.84–1.64)	1.33 (0.95–1.86)	1.28 (1.00–1.63)	1.27 (0.61–2.63)	0.95 (0.48–1.86)

	Influenza (5111 hospitalized patients during 2015–2019)					
	Death	HF	CKD	CRD	MI	Stroke
Number of events	247	353	201	525	85	98
T1D vs. T2D	1.62 (1.12–2.33)	0.90 (0.50–1.62)	0.95 (0.56–1.61)	1.02 (0.69–1.51)	1.33 (0.50–3.52)	1.45 (0.55–3.81)
Age (per 10-year increase)	1.79 (1.65–1.95)	1.29 (1.15–1.46)	0.86 (0.75–0.97)	1.08 (0.99–1.18)	1.21 (0.95–1.52)	1.32 (1.06–1.64)
Female sex	0.93 (0.81–1.08)	1.06 (0.86–1.31)	1.24 (0.93–1.65)	1.08 (0.90–1.28)	0.82 (0.52–1.29)	0.91 (0.60–1.37)
HF	1.60 (1.37–1.87)	4.68 (3.62–6.05)	1.20 (0.87–1.66)	2.99 (2.44–3.66)	1.21 (0.75–1.94)	1.11 (0.71–1.76)
CKD	1.37 (1.14–1.63)	1.35 (1.06–1.74)	7.76 (5.70–10.58)	2.53 (2.08–3.06)	1.25 (0.72–2.18)	1.32 (0.78–2.23)
Ischemic heart disease	0.86 (0.74–1.00)	1.45 (1.16–1.82)	1.17 (0.86–1.58)	1.29 (1.07–1.55)	3.25 (1.98–5.33)	1.27 (0.83–1.93)
Stroke	1.25 (1.07–1.45)	0.96 (0.76–1.21)	1.12 (0.82–1.53)	1.03 (0.85–1.25)	1.15 (0.72–1.83)	1.94 (1.29–2.92)
Peripheral artery disease	1.30 (1.06–1.59)	1.32 (0.99–1.77)	1.62 (1.13–2.30)	1.39 (1.11–1.76)	1.58 (0.90–2.77)	0.88 (0.46–1.67)
Pneumonia	1.32 (1.15–1.53)	1.27 (1.03–1.58)	0.99 (0.74–1.32)	1.19 (1.00–1.42)	0.82 (0.51–1.30)	1.06 (0.70–1.61)
COPD	1.19 (0.99–1.43)	1.27 (0.99–1.63)	1.23 (0.86–1.75)	1.24 (1.01–1.53)	0.88 (0.48–1.62)	1.29 (0.77–2.17)
Obesity	1.18 (0.95–1.46)	1.29 (0.98–1.70)	1.00 (0.70–1.43)	1.13 (0.90–1.42)	1.37 (0.76–2.47)	0.89 (0.47–1.68)
Rheumatologic disease	1.34 (1.12–1.62)	1.13 (0.86–1.49)	1.47 (1.05–2.06)	1.26 (1.02–1.57)	0.89 (0.47–1.72)	1.58 (0.93–2.66)

Diabetes patients with type 1 (T1D) or type 2 diabetes (T2D) hospitalized for Covid-19 or influenza. Risks were estimated with age, sex, HF, CKD, ischemic heart disease, stroke, peripheral artery disease, previous pneumonia, COPD, obesity and rheumatologic disease as covariates and presented as adjusted hazard ratio values including 95% confidence interval

There were no significant associations between diabetes type and risk of any cardiovascular or renal outcome in either influenza or COVID-19. HF appeared strongly associated with subsequent risk of CKD, and vice versa. Similarly, previous ischemic heart disease was associated with higher risk of MI, and the same applied for stroke. There were otherwise no significant associations for the cardiorenal outcomes in COVID, apart from obesity being associated with a higher risk of HF.

#### Mortality and morbidity associated with COVID-19 vs. influenza

Mortality risks were higher in T1D patients hospitalized with COVID-19 vs. influenza (HR 2.44, 95% CI 1.60–3.72), whereas no significant risk differences for cardiorenal outcomes were observed, Table 4. Also, T2D patients hospitalized with COVID-19 were at higher risk of death compared with those hospitalized with influenza (HR 2.81, 95% CI 2.59–3.06), whereas the risk of both MI and HF was lower. Obesity diagnosis was only significantly associated with heart failure in T2D. HF and CKD were associated with increased mortality in both T1D and T2D. Ischemic heart disease was associated with increased risk of HF in T2D, but not increased mortality, for details see Additional file 1: Table S6 and S7.

**Table 4:** Fully adjusted risks (hazard ratios) of death within 28 days and cardiorenal complications within 1 year after admission,  in COVID-19 vs. influenza

	Type 1 diabetes (677 hospitalized patients)					
	Death	HF	CKD	CRD	MI	Stroke
Number of events	73	22	28	47	8	9
COVID-19 vs. influenza	2.44 (1.60–3.72)	0.79 (0.31–1.99)	0.66 (0.28–1.55)	0.70 (0.37–1.31)	0.58 (0.13–2.56)	0.85 (0.22–3.28)

	Type 2 diabetes (15,439 hospitalized patients)					
	Death	HF	CKD	CRD	MI	Stroke
Number of events	2199	560	407	920	140	186
COVID-19 vs. influenza	2.81 (2.59–3.06)	0.67 (0.56–0.80)	0.98 (0.80–1.20)	0.78 (0.68–0.89)	0.65 (0.46–0.92)	0.85 (0.63–1.15)

Diabetes patients with type 1 (T1D) or type 2 diabetes (T2D) hospitalized for Covid-19 or influenza. Risks were estimated with age, sex, HF, CKD, ischemic heart disease, stroke, peripheral artery disease, previous pneumonia, COPD, obesity and rheumatologic disease as covariates and presented as adjusted hazard ratio values including 95% confidence interval

#### Subgroup analysis

We did a subgroup analysis of patients free of previous cardiovascular and renal disease at baseline. 5580 such patients were identified in the COVID-19 cohort (183 T1D, 5937 T2D) and 1792 patients in the influenza cohort (136 T1D, 1656 T2D). There were very few cardiovascular and renal events in this subgroup, for example only 20 CRD events in T2D patients hospitalized with COVID-19. No meaningful statistical comparisons could be made between groups. The number of outcomes are presented in Additional file 1: Table S8.

## Conclusions

### Main findings

In our nationwide cohorts of patients with diabetes who were hospitalized due to COVID-19, we found that almost 20% of the patients died within 28 days. T1D patients were at greater risk of dying within 28 days compared to T2D patients after both COVID-19 and influenza hospitalization. The risk of cardiorenal disease events after COVID-19 hospitalization was higher for T1D compared with T2D, and this was driven by HF. We made several key observations.

First, age- and sex-adjusted risks of death and HF were significantly increased for T1D relative to T2D in COVID-19 by about 80% and 160%, respectively. In influenza, there was an 80% greater risk of dying for T1D patients compared with T2D patients. With full adjustment for prior comorbidities, the association of increased mortality in T1D vs. T2D was still significant in both COVID-19 and influenza, but was no longer significant for HF. There were also no significant associations for stroke, MI and CKD. This implies that while T1D does exhibit a generally increased net risk of unfavourable outcomes, this may largely be mediated by the presence of other comorbidities. This is further supported by the fact that very few events occurred in a subgroup free of cardiovascular and renal disease.

Second, prior ischemic heart disease was not associated with higher mortality in neither COVID-19 nor influenza, which seems surprising. Possibly, secondary preventive medications and lifestyle intervention had a protective effect in these patients. The lack of increased mortality risk with obesity is also an unexpected finding. Notably, as prior conditions were captured by diagnoses from hospitals, there is under-reporting of obesity, which may lead to selection bias.

Third, COVID-19 relative to influenza appears to confer greater mortality in both T2D and T1D, though to a somewhat greater extent among T2D patients.

Our findings of increased age- and sex-adjusted COVID-19 mortality in T1D vs. T2D patients seem to differ slightly from some previous research findings. Rawshani et al.[9] examined risks in COVID-19 for T1D and T2D separately, in a Swedish cohort based on data from the National Diabetes Register (NDR), whereas no comparisons were made between T1D and T2D directly. There was a higher risk of death for T1D compared to controls seen in age- and sex-adjusted analyses, but this did not remain after full adjustment for other covariates such as education, income, treatment and comorbidities. McGurnaghan and colleagues[10] found similar risks of COVID-19-related death in T1D and T2D in their Scottish nationwide cohort of some 320,000 patients with diabetes. Body mass index (BMI) was not significantly associated with the risk of having fatal or critical care unit-treated COVID-19. This is unexpected given the well-established higher risk of severe disease and mortality in general COVID-19 populations conferred by obesity [11, 12]. Prior studies in diabetes cohorts likewise did not show that obesity is associated with a higher risk of severe disease outcomes [13]. We did not find obesity to be linked with any adverse outcomes, but our results should be interpreted with caution due to lack of data on BMI in our study and limited coverage of obesity in the utilized hospital-based patient register.

Previous studies have shown advanced age[14] and male sex[15] to be important risk factors of severe COVID-19 in general populations, including death. In our cohort, we found this to hold true as well. Pre-existing cardiorenal disease also appeared to contribute to excess mortality, as did previous pulmonary disease.

Surprisingly,  we found no significant association between prior ischemic heart disease and mortality in either COVID-19 or influenza. This finding could be due to intense secondary preventive medication regimen after a diagnosis of myocardial infarction or angina pectoris, although one would also expect patients suffering a stroke to similarly be treated with drugs such as low-dose aspirin and statins. Nonetheless, it is quite plausible that the former patient group is more intensively treated with such drugs. It is also interesting to note that the risk of non-fatal MI and HF in T2D was about 30% lower in COVID-19 as compared with influenza. Influenza has been implicated in the pathogenesis of acute myocardial infarction[16] and this could also result in increased rates of heart failure due to chronic myocardial ischemia.

In our study, we did not have a control group of patients without diabetes with which to compare the risk of death. Overall, 60-day mortality was 19.0% for T1D and 20.9% for T2D. This was higher in 2020 (21.8% T1D, 23.6% T2D) than in 2021 (12.6% T1D, 16.2% T2D; see Additional file 1: Table S4). Strålin et al. [17] examined all patients hospitalized with COVID-19 in Sweden between March and November 2020. They found an average 60-day mortality of 17.4%, decreasing from 24.7% in March 2020 during the first wave to 10.4% later on, in July–September 2020. Data from the Swedish National Board of Health and Welfare showed a slight increase in mortality during the autumn months of 2021 (for example around 10% in October) compared to the summer months, as expected in the case of a respiratory pathogen [18]. It therefore appears that the mortality in our cohort is comparable to, or slightly higher than, that of the general COVID-19 hospitalized population in Sweden. In this regard, it is important to note the findings of Bergman and colleagues, who showed that among patients hospitalized with COVID-19, around 25% had diabetes (as compared to 6% in the general population) [19]. Incidence of hospitalization among patients with T2D appears to be higher than that in the general population, while among T1D it appears to be similar to the general population. While we did not have a control group to compare with, considering the age category of 70–79 years in the general population of Sweden, there were 20,005 admissions from the beginning of the pandemic until June 3, 2022, comprising around 1.9% of the population in this age span[20, 21]. This may be compared with our T2D cohort which had a mean age of 70 years and in which 3.3% were hospitalized. For the age category of 50–59 years, there were 14,014 admissions, comprising around 1.1% of the general population at these ages. In our T1D cohort, mean age was 58 years and around 0.9% were hospitalized.

Our results indicate a trend of decreasing mortality of COVID-19 over time, and even more so after the introduction of the nationwide mass vaccination campaign during the spring of 2021. In addition to the protective effect of vaccination, this trend can probably be explained by improved therapeutic options such as treatment with corticosteroids and monoclonal antibodies. It is also possible that the individuals who died during the first wave were generally frailer and more susceptible to severe COVID-19.

To the best of our knowledge, our study is the first to compare outcomes of COVID-19 and influenza in T1D and T2D. Piroth et al.[22] previously compared a general population of around 89,000 COVID-19 and 45,000 seasonal influenza patients who were admitted to hospital and found a relative risk of death of 2.9 for COVID-19 cases vs. influenza. In basic agreement with these findings, we found an approximately 2.8-fold increase in mortality with COVID-19 compared to influenza among patients with T2D and also a 2.4-fold higher risk in T1D. This would suggest that patients with T2D are somewhat more affected by COVID-19 in comparison to influenza than T1D.

## Strengths and limitations

The strengths of this study include the nationwide coverage of virtually all patients with T1D and T2D in Sweden during the time period of the study. Collecting data on influenza from several years prior to 2020–2021 allowed us to compare the characteristics of the COVID-19 pandemic with those of the previous seasonal influenza pandemics. Furthermore, our data encompassed several of the COVID-19 pandemic waves, providing a more complete and dynamic picture of the whole pandemic, illustrating a generally decreased risk of complications as vaccinations were administered and therapeutic options improved.

Our study also has several limitations. We did not have access to data on HbA1c and BMI, so we were not able to adjust for these factors in our analysis. Hyperglycaemia at hospital admission has been associated with worse outcomes, although this has not been shown for HbA1c levels [13]. The diagnosis of obesity recorded in the NPR is likely to provide an incomplete coverage of patients with overweight and obesity as only hospital care will lead to the diagnosis being recorded. Furthermore, it does not allow for stratification into overweight and grades of obesity. The ICD-10 codes of CKD and HF may also be underrepresented as diagnosis in primary care will not appear in the NPR, although clinically significant events such as heart failure hospitalization will be recorded. We also did not have access to data on individual vaccine status and index hospitalization use of specific COVID-19 therapies, and variations in vaccine uptake and varying use of specific therapies may also have contributed to differences in outcomes. The results may also not be generalizable to populations in other countries as there were no data on the ethnic composition of the patient population. The relatively low number of hospitalizations and events in T1D limit the statistical power of the outcomes in the T1D group. Finally, diabetes patients dying outside of hospital, for example in nursing homes, were not recorded in the NPR and could therefore not be included in our analyses.

In summary, this study shows that diabetes patients hospitalized with COVID-19 had more than twice as high mortality risk compared to influenza. Moreover, the age- and sex-adjusted mortality risk for T1D patients was higher compared to those with T2D, both when hospitalized for COVID-19 and influenza. COVID-19 was associated with high risk of death in patients with diabetes, especially those with T1D, highlighting the importance of preventive measures such as vaccination and personal protection.

## Supplementary Information

**Additional file 1: Table S1.** ICD-10 diagnosis codes of baseline variables. **Table S2.** ICD-10 diagnosis codes of outcomes. **Table S3. **Follow-up times for outcomes according to viral disease and diabetes type. **Table S4. **28-day and 60-day mortality in COVID-19 during 2020 and 2021. **Table S5. **Number of events and event rates of death within 28 days and cardiorenal complications within up to 1 year after index date. Data are n (%; events/100 patient-years). **Table S6. **Fully adjusted risk of death within 28 days and cardiorenal complications within up to 1 year after index date, in relation to baseline conditions in T1D. **Table S7. **Fully adjusted risk of death within 28 days and cardiorenal complications within up to 1 year after index date, in relation to baseline conditions in T2D. **Table S8.** Number of outcomes in patients without previous cardiovascular or renal disease in T1D and T2D, in COVID-19 and influenza.

## Data Availability

The datasets generated during and/or analyzed during the current study are not publicly available due to restrictions that apply under the approval granted by the registry holder for the current study.

## Abbreviations

BMI: Body mass index
CKD: Chronic kidney disease
CoDR: Cause of Death Register
COPD: Chronic obstructive pulmonary disease
COVID-19: Coronavirus disease 2019
CRD: Cardiorenal disease
HF: Heart failure
HR: Hazard ratio
MI: Myocardial infarction
NPR: National Patient Register
PDR: Prescribed Drug Register
PIN: Personal identification number
SARS-CoV-2: Severe acute respiratory syndrome coronavirus 2
T1D: Type 1 diabetes
T2D: Type 2 diabetes
WHO: World Health Organization
