# Supplementary material for: Patients with type 1 and type 2 diabetes hospitalized with COVID-19 in comparison with influenza: mortality and cardiorenal complications assessed by nationwide Swedish registry data
Source: Cardiovasc Diabetol. 2022 Dec 15;21:282. doi: 10.1186/s12933-022-01719-x (PMC9753889; doi:10.1186/s12933-022-01719-x)
Supplement: Supplementary file 1 — Additional file 1: Table S1. ICD-10 diagnosis codes of baseline variables. Table S2. ICD-10 diagnosis codes of outcomes. Table S3. Follow-up times for outcomes according to viral disease and diabetes type. Table S4. 28-day and 60-day mortality in COVID-19 during 2020 and 2021. Table S5. Number of events and event rates of death within 28 days and cardiorenal complications within up to 1 year after index date. Data are n (%; events/100 patient-years). Table S6. Fully adjusted risk of death within 28 days and cardiorenal complications within up to 1 year after index date, in relation to baseline conditions in T1D. Table S7. Fully adjusted risk of death within 28 days and cardiorenal complications within up to 1 year after index date, in relation to baseline conditions in T2D. Table S8. Number of outcomes in patients without previous cardiovascular or renal disease in T1D and T2D, in COVID-19 and influenza. [file 12933_2022_1719_MOESM1_ESM.docx]

**Additional file 1: Table S1. ICD-10 diagnosis codes of baseline variables.**

| **Disease** | **ICD-10 code** | **Surgical code** |
| --- | --- | --- |
| COVID-19 | U07.1, U07.2 |  |
| Influenza | J09.9, J10-J11 |  |
| **Ischemic heart disease** |  |  |
| Myocardial infarction | I21-I22, I25.2, I25.6 |  |
| Unstable angina | I20.0 |  |
| PCI with stent |  | FNG |
| CABG |  | FNA-FNE |
| Angina pectoris | I20.1, I20.8, I20.9, I25.1, I25.5 |  |
| **Heart failure (total)** | I50, I11.0, I13.0, I13.2 |  |
| Heart failure | I50 |  |
| Heart failure - hypertensive | I11.0, I13.0, I13.2 |  |
| **CKD (total)** | N17-N19, I12.0-I12.9, I13.1, I13.2, N08.3, E10.2, E11.2, E12.2, E13.2, E14.2, Z49, Z99.2 |  |
| CKD - Acute | N17 |  |
| CKD - Chronic | N18 |  |
| CKD - Unspecified | N19 |  |
| CKD - Diabetic | E10.2, E11.2, E12.2, E13.2, E14.2, N08.3 |  |
| CKD - Hypertensive | I12.0-I12.9, I13.1, I13.2 |  |
| CKD - Dialysis | Z49, Z99.2 |  |
| Atrial fibrillation | I48 |  |
| Stroke | I60-I66, G45 |  |
| Hemorrhagic | I60-I62 |  |
| Ischemic | I63 |  |
| Peripheral artery disease | I70.2, I73.9, I74.2-9 |  |
| Dialysis | Z49, Z99.2 |  |
| Cancer | C00-C99 |  |
| COPD | J44 |  |
| Pneumonia | J10-J18 |  |
| Obesity | E66 |  |
| Rheumatologic disease | M05-M14, M30-M36, M790 |  |

PCI, percutaneous coronary intervention. CABG, coronary artery bypass graft. Surgical codes are according to the NOMESCO Classification of Surgical Procedures.

**Additional file 1: Table S2. ICD-10 diagnosis codes of outcomes.**

| **Outcome** | **ICD-10 code** |
| --- | --- |
| Myocardial infarction | I21-I22 |
| Heart failure | I50, I11.0, I13.0, I13.2 |
| CKD | N17-N19, I12.0-I12.9, I13.1, I13.2, N08.3, E10.2, E11.2, E12.2, E13.2, E14.2, Z49, Z99.2 |
| Stroke | I60-I64 |

**Additional file 1: Table S3. Follow-up times for outcomes according to viral disease and diabetes type.**

|  | **Covid-19 –Diabetes (n=11,005)** | **Covid-19- T1D (n=373)** | **Covid-19 – T2D (n=10,632)** | **Influenza –Diabetes (n=5,111)** | **Influenza – T1D (n=304)** | **Influenza – T2D (n=4,807)** |
| --- | --- | --- | --- | --- | --- | --- |
| **Death** | | | | | | |
| Months of follow-up per individual (mean) | 7.41 | 8.10 | 7.38 | 10.31 | 10.57 | 10.30 |
| Person-years (sum) | 19,391 | 962 | 18,429 | 4,388 | 268 | 4,12 |
| **HF** | | | | | | |
| Months of follow-up per individual (mean) | 7.13 | 7.94 | 7.09 | 9.91 | 10.29 | 9.89 |
| Person-years (sum) | 18,650 | 943 | 17,707 | 4,217 | 260 | 3,957 |
| **CKD** | | | | | | |
| Months of follow-up per individual (mean) | 7.16 | 7.80 | 7.13 | 10.10 | 10.25 | 10.09 |
| Person-years (sum) | 18,750 | 926 | 17,824 | 4,297 | 259 | 4,037 |
| **CRD** | | | | | | |
| Months of follow-up per individual (mean) | 6.91 | 7.66 | 6.87 | 9.72 | 10.00 | 9.70 |
| Person-years (sum) | 18,073 | 910 | 17,164 | 4,136 | 253 | 3,883 |
| **MI** | | | | | | |
| Months of follow-up per individual (mean) | 7.07 | 7.52 | 7.05 | 10.22 | 10.45 | 10.21 |
| Person-years (sum) | 6,473 | 234 | 6,239 | 4,349 | 264 | 4,084 |
| **Stroke** | | | | | | |
| Months of follow-up per individual (mean) | 7.05 | 7.52 | 7.03 | 10.23 | 10.44 | 10.21 |
| Person-years (sum) | 6,454 | 233 | 6,22 | 4,351 | 264 | 4,087 |

**Additional file 1: Table S4. 28-day and 60-day mortality in COVID-19 during 2020 and 2021.**

|  | **Covid-19 - Diabetes** | **Covid-19 – T1D** | **Covid-19 – T2D** |
| --- | --- | --- | --- |
| **2020** | **n=6,947** | **n=262** | **n=6,685** |
| Deaths within 28 days, n (%) | 1,461 (21.0) | 51 (19.5) | 1,410 (21.1) |
| Deaths within 60 days, n (%) | 1,638 (23.6) | 57 (21.8) | 1,581 (23.6) |
| **2021** | **n=4,058** | **n=111** | **n=3,947** |
| Deaths within 28 days, n (%) | 564 (13.9) | 12 (10.8) | 552 (14.0) |
| Deaths within 60 days, n (%) | 655 (16.1) | 14 (12.6) | 641 (16.2) |

**Additional file 1: Table S5. Number of events and event rates of death within 28 days and cardiorenal complications within up to 1 year after index date. Data are n (%; events/100 patient-years).**

|  | **Death** | **HF** | **CKD** | **CRD** | **MI** | **Stroke** |
| --- | --- | --- | --- | --- | --- | --- |
| **COVID-19** |  | | | | | |
| T1D | 63 (16.9; 247) | 9 (2.4; 4) | 8 (2.1; 4) | 16 (4.3; 7) | 3 (0.8; 1) | 4 (1.1; 2) |
| T2D | 1,962 (18.5; 273) | 220 (2.1; 4) | 226 (2.1; 4) | 426 (4.0; 7) | 60 (0.5; 1) | 93 (0.9; 2) |
| **Influenza** |  | | | | | |
| T1D | 10 (3.3; 44) | 13 (4.3; 5) | 20 (6.6; 8) | 31 (10.2; 12) | 5 (1.6; 2) | 5 (1.6; 2) |
| T2D | 237 (4.9; 67) | 340 (7.1; 9) | 181 (3.8; 5) | 494 (10.3; 12) | 80 (1.7; 2) | 93 (1.9; 2) |

**Additional file 1: Table S6. Fully adjusted risk of death within 28 days and cardiorenal complications within up to 1 year after index date, in relation to baseline conditions in T1D.**

| **T1D**  n=677 | **Death**  n=73 | **HF**  n=22 | **CKD**  n=28 | **CRD**  n=47 | **MI**  n=8 | **Stroke**  n=9 |
| --- | --- | --- | --- | --- | --- | --- |
| COVID-19 vs influenza | 2.44 (1.60-3.72) | 0.79 (0.31-1.99) | 0.66 (0.28-1.55) | 0.70 (0.37-1.31) | 0.58 (0.13-2.56) | 0.85 (0.22-3.28) |
| Age (per 10-year increase) | 1.92 (1.61-2.28) | 2.41 (1.55-3.75) | 1.00 (0.75-1.35) | 1.39 (1.08-1.78) | N/A | N/A |
| Female sex | 0.91 (0.62-1.34) | 0.43 (0.16-1.19) | 1.03 (0.46-2.26) | 0.67 (0.36-1.27) | N/A | N/A |
| HF | 1.69 (1.06-2.70) | 1.57 (0.55-4.50) | 1.16 (0.43-3.10) | 1.68 (0.80-3.53) | N/A | N/A |
| CKD | 1.64 (1.07-2.51) | 1.34 (0.48-3.71) | 8.49 (3.32-21.71) | 3.28 (1.69-6.37) | N/A | N/A |
| Ischemic heart disease | 0.68 (0.43-1.07) | 1.21 (0.44-3.33) | 0.95 (0.35-2.55) | 0.84 (0.41-1.72) | N/A | N/A |
| Stroke | 1.86 (1.23-2.81) | 1.24 (0.44-3.54) | 2.19 (0.95-5.03) | 1.73 (0.90-3.34) | N/A | N/A |
| Peripheral artery disease | 1.38 (0.90-2.10) | 1.65 (0.65-4.16) | 2.14 (0.93-4.94) | 1.78 (0.95-3.36) | N/A | N/A |
| Pneumonia | 1.51 (1.01-2.24) | 1.24 (0.50-3.06) | 1.05 (0.48-2.32) | 1.21 (0.66-2.22) | N/A | N/A |
| COPD | 1.10 (0.63-1.93) | 1.05 (0.29-3.79) | 0.40 (0.05-3.09) | 0.90 (0.31-2.59) | N/A | N/A |
| Obesity | 0.81 (0.45-1.46) | 1.61 (0.49-5.30) | 0.27 (0.04-2.08) | 0.79 (0.30-2.11) | N/A | N/A |
| Rheumatologic disease | 0.98 (0.58-1.67) | 1.61 (0.55-4.71) | 0.91 (0.30-2.77) | 1.26 (0.59-2.70) | N/A | N/A |

Data are hazard ratio values including 95 % confidence interval. N/A, not applicable due to small number of events.

**Additional file 1: Table S7. Fully adjusted risk of death within 28 days and cardiorenal complications within up to 1 year after index date, in relation to baseline conditions in T2D.**

| **T2D**  n=15,439 | **Death**  n=2,199 | **HF**  n=560 | **CKD**  n=407 | **CRD**  n=920 | **MI**  n=140 | **Stroke**  n=186 |
| --- | --- | --- | --- | --- | --- | --- |
| COVID-19 vs influenza | 2.81 (2.59-3.06) | 0.67 (0.56-0.80) | 0.98 (0.80-1.20) | 0.78 (0.68-0.89) | 0.65 (0.46-0.92) | 0.85 (0.63-1.15) |
| Age (per 10 year increase) | 1.94 (1.87-2.01) | 1.43 (1.30-1.57) | 1.04 (0.95-1.14) | 1.22 (1.14-1.30) | 1.26 (1.06-1.49) | 1.31 (1.13-1.52) |
| Female sex | 0.82 (0.77-0.89) | 0.97 (0.82-1.16) | 1.05 (0.85-1.28) | 0.97 (0.85-1.11) | 0.89 (0.63-1.27) | 0.93 (0.69-1.26) |
| HF | 1.34 (1.24-1.46) | 6.28 (5.07-7.78) | 1.24 (0.98-1.58) | 3.29 (2.81-3.85) | 1.37 (0.93-2.02) | 1.14 (0.80-1.64) |
| CKD | 1.52 (1.39-1.66) | 1.48 (1.22-1.79) | 6.82 (5.49-8.48) | 2.76 (2.38-3.19) | 1.29 (0.82-2.02) | 1.40 (0.94-2.08) |
| Ischemic heart disease | 1.01 (0.93-1.08) | 1.35 (1.13-1.62) | 1.00 (0.81-1.24) | 1.16 (1.01-1.33) | 2.63 (1.82-3.81) | 1.23 (0.90-1.69) |
| Stroke | 1.17 (1.08-1.26) | 0.92 (0.76-1.11) | 1.02 (0.81-1.29) | 0.97 (0.83-1.13) | 1.27 (0.88-1.85) | 2.04 (1.50-2.77) |
| Peripheral artery disease | 1.35 (1.22-1.50) | 1.18 (0.93-1.50) | 1.33 (0.99-1.78) | 1.17 (0.96-1.42) | 1.10 (0.65-1.88) | 0.88 (0.52-1.49) |
| Pneumonia | 1.34 (1.25-1.45) | 1.22 (1.02-1.45) | 1.22 (0.98-1.51) | 1.24 (1.08-1.43) | 0.86 (0.58-1.25) | 1.02 (0.74-1.42) |
| COPD | 1.12 (1.02-1.23) | 1.34 (1.10-1.64) | 1.12 (0.86-1.47) | 1.25 (1.06-1.47) | 0.80 (0.48-1.34) | 1.05 (0.69-1.61) |
| Obesity | 1.09 (0.99-1.21) | 1.35 (1.09-1.66) | 1.14 (0.89-1.45) | 1.22 (1.04-1.44) | 1.08 (0.67-1.72) | 0.68 (0.42-1.09) |
| Rheumatologic disease | 1.06 (0.96-1.17) | 1.12 (0.90-1.39) | 1.44 (1.12-1.83) | 1.26 (1.07-1.49) | 1.12 (0.69-1.83) | 1.28 (0.84-1.94) |

Data are hazard ratio values including 95 % confidence interval.

**Additional file 1: Table S8. Number of outcomes in patients without previous cardiovascular or renal disease in T1D and T2D, in COVID-19 and influenza.**

| **Covid-19**  n=5,580 | **Death** | **HF** | **CKD** | **CRD** | **MI** | **Stroke** |
| --- | --- | --- | --- | --- | --- | --- |
| T1D | 12 | 0 | 1 | 1 | 0 | 0 |
| T2D | 538 | 2 | 18 | 20 | 5 | 8 |
| **Influenza**  n=1,792 | **Death** | **HF** | **CKD** | **CRD** | **MI** | **Stroke** |
| T1D | 2 | 0 | 0 | 0 | 0 | 0 |
| T2D | 45 | 2 | 4 | 6 | 2 | 4 |
